# Antioxidant-upregulated mesenchymal stem cells reduce inflammation and improve fatty liver disease in diet-induced obesity

**DOI:** 10.1186/s13287-019-1393-8

**Published:** 2019-09-02

**Authors:** Cleyton C. Domingues, Nabanita Kundu, Yana Kropotova, Neeki Ahmadi, Sabyasachi Sen

**Affiliations:** 1 Department of Medicine, The George Washington University, Washington, DC, USA; 2 School of Medicine and Health Science, 2300 I Street NW, Washington, DC, 20037 USA

**Keywords:** Antioxidants, Adipose tissue-derived stem cells, Diabetes, Obesity, Inflammation

## Abstract

**Background:**

The incidence of obesity and diabetes is increasing rapidly. Optimal management is still elusive. Obesity associated with type 2 diabetes is known to cause adipose tissue inflammation, increase oxidative stress, and cause white fat hyperplasia and mitochondrial dysfunction. In this study, we investigated whether mitochondrial and cytosolic antioxidant-upregulated mesenchymal stem cell (MSC) delivery reduces oxidative stress and subsequently improves glucose tolerance, reduce systemic inflammation, and improves fatty liver disease in diet-induced obese (DIO) mouse models.

**Methods:**

Antioxidant genes Sod2 (mitochondrial) and catalase (cytosolic) or null (control) were upregulated in human adipose tissue-derived MSCs using adenoviral constructs. Modified MSCs were then delivered intraperitoneally into mice that were fed a 45% or 60% high-fat diet (HFD), and animals were followed for 4 weeks.

**Results:**

Over 4 weeks, body weight remained stable; however, we noted a significant reduction in liver fat content by histological analysis and liver triglyceride assay. Triglyceride assay (*p* < 0.01) confirmed reduced liver fat accumulation in animals that received either Sod2- or Cat-MSCs. There was a lower plasma level of inflammatory marker TNFα, measured in mice that were fed either 45% or 60% HFD and received Sod2- or Cat-MSCs, indicating reduced systemic inflammation. Ucp1 mRNA was upregulated approximately 100–1000-fold for omental fat and 10–100-fold for pericardial fat compared to the Null-MSC-receiving group. Pcgc1a and Prdm16 mRNA upregulation was also noted particularly for pericardial fat. Glucose tolerance showed a positive improvement trend with a lower area under the curve (AUC) values for both Sod2- and Cat-MSCs groups in comparison to control. For mice fed with 60% HFD and that received Sod2-MSCs, glucose levels were significantly lower than control (*p < 0.05) at a time point of 60 min in the glycemic curve during glucose tolerance test.

**Conclusion:**

Reduction of oxidative stress post-antioxidant-upregulated MSC delivery, intraperitoneally, reduces systemic inflammation and fat accumulation in the liver. There is evidence of an increase in browning of white adipose tissue depots with concomitant improvement of glucose tolerance in a weight-independent fashion. Antioxidant-upregulated MSC delivery may be a safe yet effective therapy for obesity and prediabetes and improves related complication such as non-alcoholic fatty liver disease.

**Electronic supplementary material:**

The online version of this article (10.1186/s13287-019-1393-8) contains supplementary material, which is available to authorized users.

## Introduction

Obesity and type 2 diabetes (T2D) are complex metabolic chronic diseases where a cure has been elusive. Although many therapies that improve the management of obesity and diabetes are available, improvement of diabetes- and obesity-related complications still remains a challenge for many patients. The number of people with diabetes is increasing globally, and the number of new cases has been growing both in developing and developed countries [1, 2].

Diabetes and prediabetes are also directly associated with cardiovascular diseases (CVD) [3, 4], and obesity plays an important role in the development of diabetes and resultant CVD. Obesity associated with T2D is nowadays prevalent in both adolescents and adults [3, 5]. Diabetes and obesity are known to cause adipose inflammation, elevated oxidative stress by increasing reactive oxygen species (ROS) accumulation, white fat hyperplasia, and mitochondrial dysfunction. These events may be inter-related, leading to insulin resistance (IR) where ROS accumulation and inflammation could be the prime driver of obesity and diabetes complications such as micro- and macrovascular complications and non-alcoholic fatty liver disease (NAFLD).

NAFLD is a highly prevalent complication of both prediabetes and diabetes and can progress to severe liver disease such as non-alcoholic steatohepatitis (NASH) [6]. Stem and progenitor cell therapy provides a novel strategy for treating obesity- and diabetes-related complications [7, 8]. Adipose tissue-derived mesenchymal stem cells (MSCs)—adult stem cells that have multipotent differentiation ability—tend to assimilate with its own source/lineage. Adipose tissue-derived MSCs are expected to home in and assimilate better with fat depots than any other mesenchymal tissue that is present in the vicinity of the point for cell delivery. Therefore, MSCs can be strong candidates to help reduce oxidative stress by delivering intracellular antioxidants to the adipose tissue depots and other adipose tissue-rich viscera such as the liver, that are present within the abdominal cavity [7, 9]**.**

Previously, we have delivered human MSCs to fat pockets in db/db leptin-resistant obese diabetic mice. Delivery (intraperitoneally) of Sod2-upregulated MSCs in obese and diabetic db/db mice not only reduced ROS (and consequently reduced inflammation) but also improved glucose tolerance and total body weight [7]. However, the molecular mechanisms for these positive effects described above have not been established yet. The effect of such therapy on systemic inflammation, for instance, still needs to be explored. MSCs have also been used to produce and improve insulin function and complications associated with diabetes and obesity, with or without genetic modification [9–11]. In animal models, MSC therapy showed positive results in diabetic nephropathy treatment that helped protect podocyte injury that was exposed to hyperglycemia, from apoptosis [12, 13]. A study using diet-induced obese (DIO) mice reported that MSC transplant, following intravenous infusion, helped to lower blood glucose and improved glucose tolerance with associated reduction of inflammatory markers in the liver [14]. In that study, 7-week-old mice were fed with a high-fat diet (60% of calories from fat) for 20 weeks before treatment was performed. In comparison, we used a similar regimen but exposed the animals to high-fat diet for a much shorter duration and used two high-fat diet regimens, 45% and 60% fat diets. We choose to use a lesser duration of HFD exposure and also a lesser percentage of fat in the diet (45%) so that the models are closer to human diet-induced obesity with lesser fat ingestion over a period of time.

We investigated whether a single intraperitoneal delivery of human adipose-derived MSCs overexpressing antioxidants either Sod2 (mitochondrial) or catalase (cytosolic) can reduce oxidative stress and promote therapeutic effects on systemic inflammation, glucose homeostasis, and diabetes-related complications such as NAFLD in different DIO mouse models. Insights into the mechanisms involved, post-cell therapy, were also explored.

## Methods

### Animals

C57BL/6J male mice (4–6 weeks old) were obtained from the Jackson Lab. Obesity, glucose intolerance, and insulin resistance were induced by feeding the mice a high-fat diet (HFD). One group was subjected to a 45% HFD (58V8, 45% of calories from fat, TestDiet, Inc.) for 14–16 weeks. The second group was subjected to a 60% HFD (58Y1, 60% of calories from fat, TestDiet, Inc.) for 8–10 weeks. The period of a particular diet was chosen in order to allow mice to reach approximately 35–40 g of body weight. All mice received a high-fat diet of a particular type and drinking water ad libitum. They were housed at 22 °C on a 12-h artificial light-dark cycle. Institutional guidelines and approved protocols were followed for all animal procedures (IACUC #A-335 and IBC #15-013, The George Washington University).

### Overexpression of Sod2 and Cat in MSCs

Human adipose-derived MSCs were commercially obtained (Lonza, catalog #PT-2501) and cultured in DMEM (1 g/L glucose) containing 10% FBS and 1% penicillin/streptomycin. Adenovirus constructs were purchased from Vector Biolabs and expanded using HEK cells. The virus was then concentrated and titrated according to a modified protocol described for Adeno-X rapid titer kit (Clontech laboratories). The adenovirus constructs were then used as a tool to overexpress the antioxidants as the genes of interest (GOI) in MSCs. MSCs were transduced using 100 multiplicities of infection (MOI) of adenovirus serotype 5 containing eGFP cassette in the plasmid. The MSC post-viral transduction infection was then cultured for 3–5 days before intraperitoneal (IP) injection into the mice. The constructs/groups were Ad-Sod2-GFP-MSCs, Ad-Cat-GFP-MSCs, and Ad-Null-GFP-MSCs. The marker gene eGFP allowed to track the transplanted MSCs in vivo.

### MSC injection and animal monitoring

1.5 million of transduced MSCs were re-suspended in PBS (0.1–0.2 mL) and delivered intraperitoneally (IP) into diet-induced obese (DIO) mice that were fed with two different diets: 45% and 60% HFD. Control mice received Ad-Null-GFP-MSCs (*n* = 3), whereas the treatment groups received Ad-Sod2-GFP-MSCs (*n* = 4) and Ad-Cat-GFP-MSCs (*n* = 3). Blood glucose level and body weight were monitored before and after cell injection up to 4 weeks. Blood glucose was assessed after the animals were fasted for 6 h by using a drop of whole blood from a tail incision and measured by a glucose meter (Contour Next Ez, Bayer).

### MSC tracking

MSC homing was tracked using fluorescence emitted by GFP in a whole-body imaging system (Xenogen Corp.) that uses whole-body laser scan. This approach allowed a non-invasive visualization of MSC bio-distribution in live animals, and the fluorescence was monitored pre- and post-MSC transplantation up to 4 weeks.

### Glucose tolerance test

Glucose tolerance test was performed after mice were fasted for 16 h and received a dose of 2 g/kg glucose (intraperitoneal (IP)). Tail vein blood glucose level was then measured at 15, 30, 60, 90, and 120 min after glucose injection. A glucose measurement was also recorded prior to glucose intraperitoneal injection. Glucose tolerance test was performed at week 4 post-MSC transplantation. Blood glucose was assessed by using a drop of whole blood from a tail incision and measured by a glucose meter (Contour Next Ez, Bayer).

### Blood processing and tissue harvesting

After 4 weeks from the time the animals received MSCs, whole blood was collected by cardiac puncture and heparin was used as an anticoagulant. Plasma was separated by centrifugation at 3500 rpm for 10 min and stored at − 80 °C for further analysis. The liver and heart were then harvested as well as fat from different fat depots such as the pericardial, omental, and subcutaneous fat. Part of the tissues collected was cut in small pieces and snap-frozen in liquid nitrogen for further analysis. Tissues were also fixed in 10% formalin for histological analysis.

### Hematoxylin and eosin staining

Hematoxylin and eosin (H&E) staining was performed as previously described [15]. Briefly, tissues were fixed in 10% formalin, subsequently embedded in paraffin, and sectioned into slices of 3 μm. The slides were then subjected to a regressive method: treated in xylene followed by absolute alcohol and alcohol 95% and rinsed with water before stained in hematoxylin for 10–15 min. Next, the batch of slides was treated in water and hydrochloric acid (1% in 70% alcohol), washed with water and followed by a treatment in ammonia water 0.25%, and rinsed with water before staining with eosin for 1–3 min. Finally, the slides were treated in alcohol 95% followed by absolute alcohol and xylene treatment.

### Gene expression analysis

Gene expression analysis of MSCs and tissues harvested from animals was performed by quantitative reverse transcriptase polymerase chain reaction (qRT-PCR). Cell or tissue total mRNA was extracted and purified using the RNeasy mini kit (Qiagen). For white adipose tissue, mRNA was extracted using the RNeasy lipid tissue mini kit (Qiagen). mRNA was then converted into cDNA by using the High-Capacity cDNA Reverse Transcription Kit (Applied Biosystems). Gene expression changes were then assessed by a CFX96 real-time qPCR system (Bio-Rad) using TaqMan Universal Master Mix II (Applied Biosystems) and inventoried probes. The gene expression analysis included antioxidants and genes associated with inflammation and mitochondrial activity. The expression of an individual gene was normalized to housekeeping 18S, and values are relative to control (Null-MSC treatment).

### Plasma concentration of TNFα

Plasma was used to quantify the level of TNFα. One hundred microliters of undiluted samples was used for the assay, and experiments were performed using a mouse TNFα solid-phase sandwich enzyme-linked immunosorbent (ELISA) kit (KMC3011, ThermoFisher). All procedures were performed according to the manufacturer’s instructions, hepatic triglycerides assay.

### Estimation of liver triglyceride

Approximately 50–100 mg of frozen liver tissues (stored at − 80 °C) was mechanically disrupted and homogenized in lysis buffer containing 5% Triton X-100 by using a tissue homogenizer. The remained insoluble cellular fragments were then removed by centrifugation at 16,000×*g* for 10 min. Extracted triglycerides were quantified using a Triglyceride Colorimetric Assay Kit (Biovision, Inc.) according to the manufacturer’s protocol. Briefly, the sample supernatant was combined with a triglyceride probe, enzyme mix, and lipase, and after 60 min, incubation in the dark absorbance was measured at 570 nm in a Synergy HT Multi-Mode Microplate Reader (BioTek Instruments, Inc.). Blank and lipase controls were subtracted from the optical density of each sample to allow for the quantification of triglycerides. Triglyceride concentrations were interpolated from the linear regression of a standard curve and normalized by the wet weight of liver tissue used in the assay.

### Immunohistochemical detection of Ucp1 in white adipose tissue

Paraffin-embedded sections of the omental fat were stained for UCP1 (Abcam Inc.: catalog number ab23841), and secondary DAB antibody was used for positive detection. Quantification of brown staining was done using ImageJ program (NIH).

### Detection of protein Sod2 in omental fat

Omental fat tissues were mechanically disrupted and homogenized in lysis buffer containing 5% Triton X-100 by using a tissue homogenizer. The remaining insoluble membrane and cellular fragments were then removed by centrifugation at 16,000×*g* for 15 min at 4 °C.

Total protein concentration was estimated using the bicinchoninic acid (BCA) assay (Pierce). Samples (5–10 μg total protein) were separated using 4–20% precast polyacrylamide gel (BioRad). Proteins from the gels were electrophoretically transferred to polyvinylidene difluoride (PVDF) membranes using a TransBlot Turbo transfer system (BioRad). The membranes were then blocked for 1 h in TBS containing 5% non-fat milk and 0.05% Tween-20, followed by overnight incubation with primary antibody anti-SOD2 (Cayman Chemical) diluted 1:1000 in TBS. After washing, the PVDF membranes were then incubated with the appropriate peroxidase-conjugated secondary antibody. Antibody was then detected using the enhanced chemiluminescent WesternSure Premium kit (LI-COR Biosciences), and image was acquired using C-DiGit Blot Scanner (LI-COR Biosciences).

### Statistical analysis

The results were analyzed using two-way ANOVA (multiple comparisons) or unpaired Student’s *t* test. Data are expressed as mean ± SD. *p* values considered statically significant were *p < 0.05, **p < 0.01, and ***p < 0.001.

## Results

### DIO mice

Body weight and blood glucose were monitored before and after MSC transplantation. After the animals were fed a high-fat diet for several weeks (8–16 weeks depending on the diet) and before MSC transplantation, the average blood glucose was 195 ± 17 mg/dL and 191 ± 41 mg/dL for 60% HFD and 45% HFD groups, respectively.

For both diets, 45% and 60% HFD mice did not develop a basal fasting hyperglycemia above 200 mg/dL. Therefore, the animals in our study can be considered closer to a prediabetic model with obesity rather than an obese hyperglycemic model as db/db mice [16]. Therefore, our model is suitable for studying diabetes-related metabolic syndrome, similar to a human disease of prediabetes [17]. The changes in body weight promoted by HFD before cell therapy are shown in Additional file 1: Figure S1. The body weight of the mice subjected to both HFD was approximately 40 g before MSC transplantation. However, no significant reduction in the body weight was observed at week 4 post-Sod2- and Cat-MSC therapy in comparison with Null-MSCs (Additional file 1: Table S1).

### MSC tracking and effect on glucose tolerance

All the adenovirus constructs used in this study were tagged with eGFP. Based on the fluorescence of those cells, this approach allowed live tracking of transduced MSCs that were transplanted in animals. We noted that transduced MSCs distributed throughout the abdominal cavity and possibly pericardial at week 1. In our previous study, the presence of GFP in omental and epidydimal fat depots of db/db mice that received eGFP MSCs was shown by immunohistochemistry and by direct laser confocal microscopy at week 2 post-cell transplantation [7]. Here, Ad-antioxidant-eGFP-MSCs remained visible up to 4 weeks post-MSC transplant as detected by laser in vivo live imaging method.

**Fig. 1:**
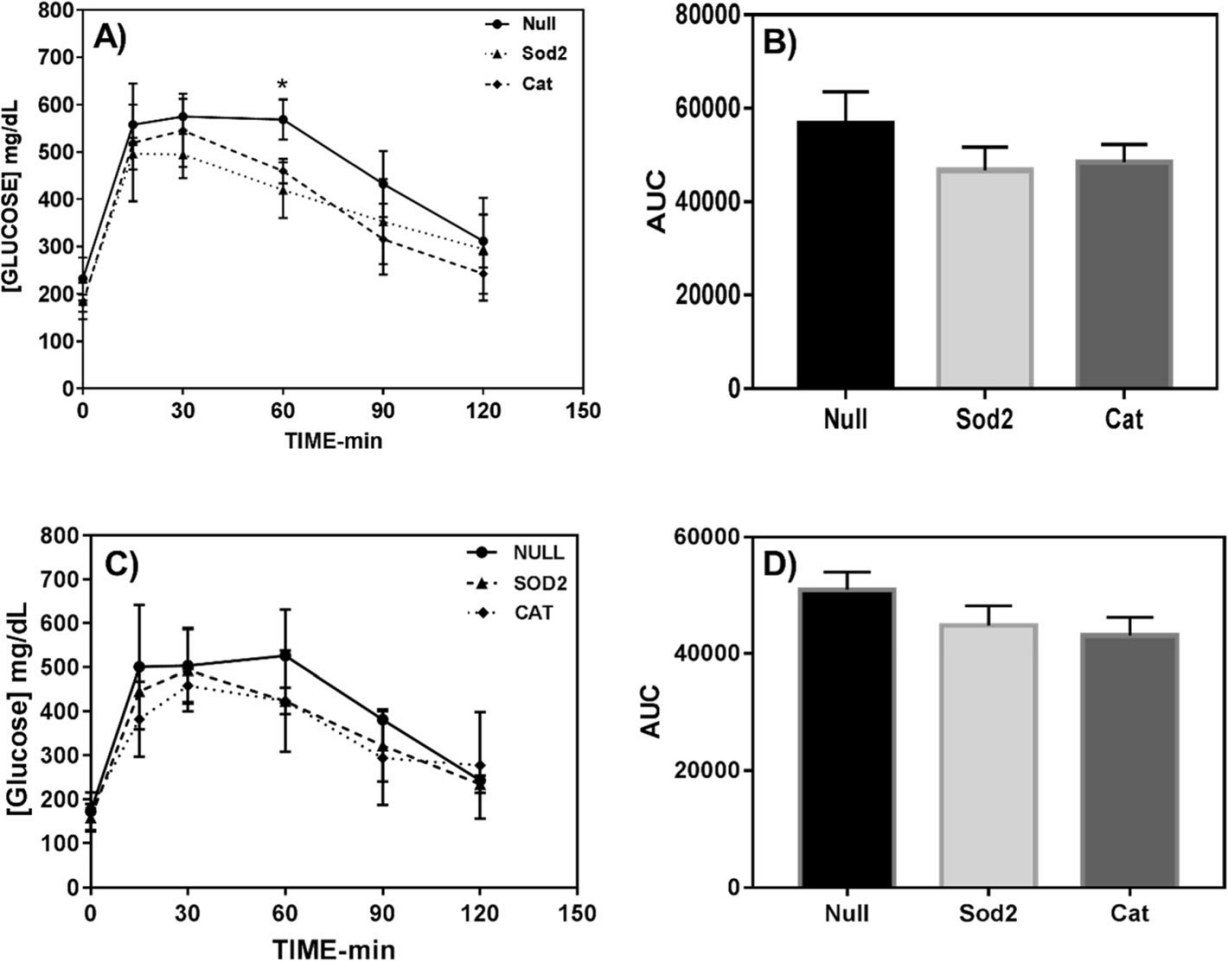
Intraperitoneal glucose tolerance test and corresponding area under the curve (AUC) at week 4 post-MSC transplantation into DIO mice. **a**, **b** 60% HFD group. **c**, **d** 45% HFD group. Animals that were fed a 60% HFD and received Sod2-MSCs showed lower AUC (**b**) in comparison with control (Null-MSCs) (*p* = 0.070), and glucose level was significantly lower than control (*p < 0.05) at a time point of 60 min in the glycemic curve (**a**). Glucose dose, 2 g/kg

The effect of MSCs overexpressing Sod2 and Cat on glucose homeostasis is shown in Fig. 1a–d. Changes in the glycemic curve were clearly observed for animals fed a 60% HFD and those received antioxidant-upregulated MSCs. A trend for a reduction in the area under the curve (AUC) (Fig. 1b) was observed for both antioxidants. Interestingly, at the time point of 60 min after glucose injection, there was a significant reduction for the group that received Sod2-MSCs (*p* < 0.05). Differences in AUC between the treatment groups and control were not statistically significant for mice fed a 45% HFD (Fig. 1c, d). However, the results showed a trend indicating lower AUC values for groups that received Sod2- and Cat-MSCs (44,808 ± 3066 and 43,050 ± 3172, respectively) in comparison with control Null-MSCs (50,968 ± 3066).

### Liver and fat histology analysis

**Fig. 2:**
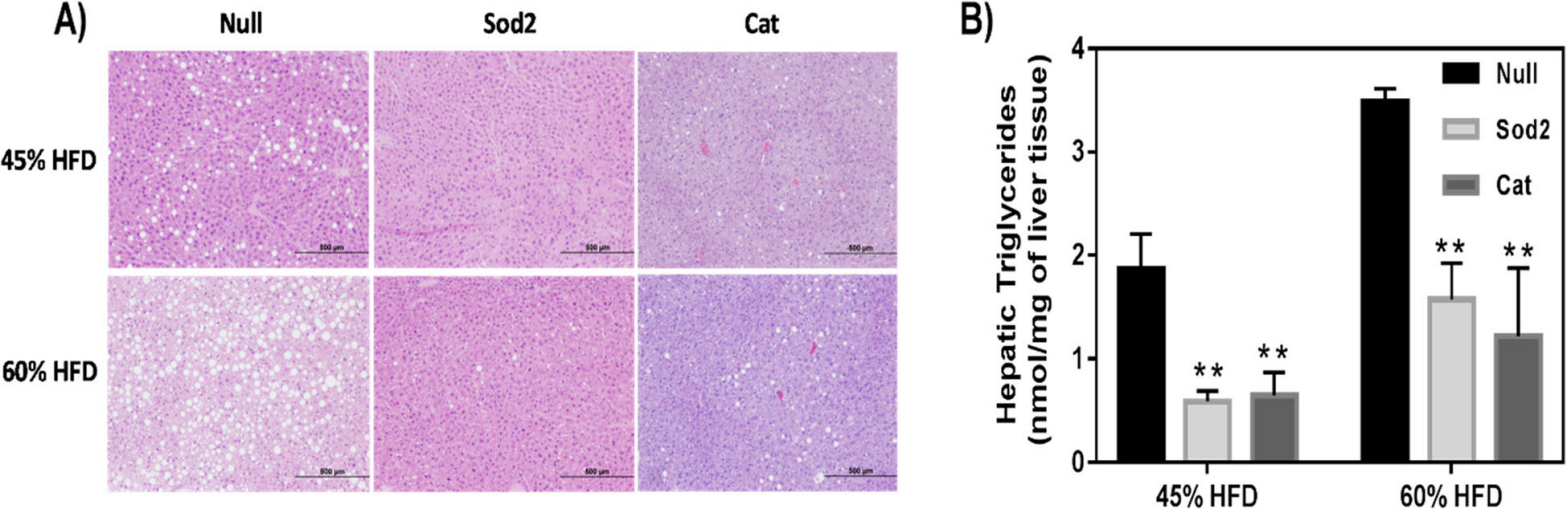
**a** Representative histology images (H&E) of the liver from DIO mice subjected to 45% and 60% HFD. The liver was harvested at week 4 post-MSC transplantation into DIO mice. Animals that received Sod2- and Cat-MSCs showed less fat accumulation in comparison with Null-MSCs (control) confirmed by **b** hepatic triglycerides quantification (**p < 0.01)

H&E staining of the liver samples harvested at week 4 post-MSC therapy showed an impressive improvement of hepatic steatosis by visual assessment. Figure 2a shows fewer fat cells in the liver from mice that received Sod2- and Cat-MSCs in comparison with control (Null-MSCs). Similar results were found for both HFD models. Additionally, small portions of liver tissue were also used to quantify triglyceride by using a commercial triglyceride quantification kit (BioVision, Cat# K622-100). The results shown in Fig. 2b confirmed a reduction in hepatic triglyceride content for the group that received Sod2- and Cat-MSCs similarly to the results noted by the histological analysis (Fig. 2a).

**Fig. 3:**
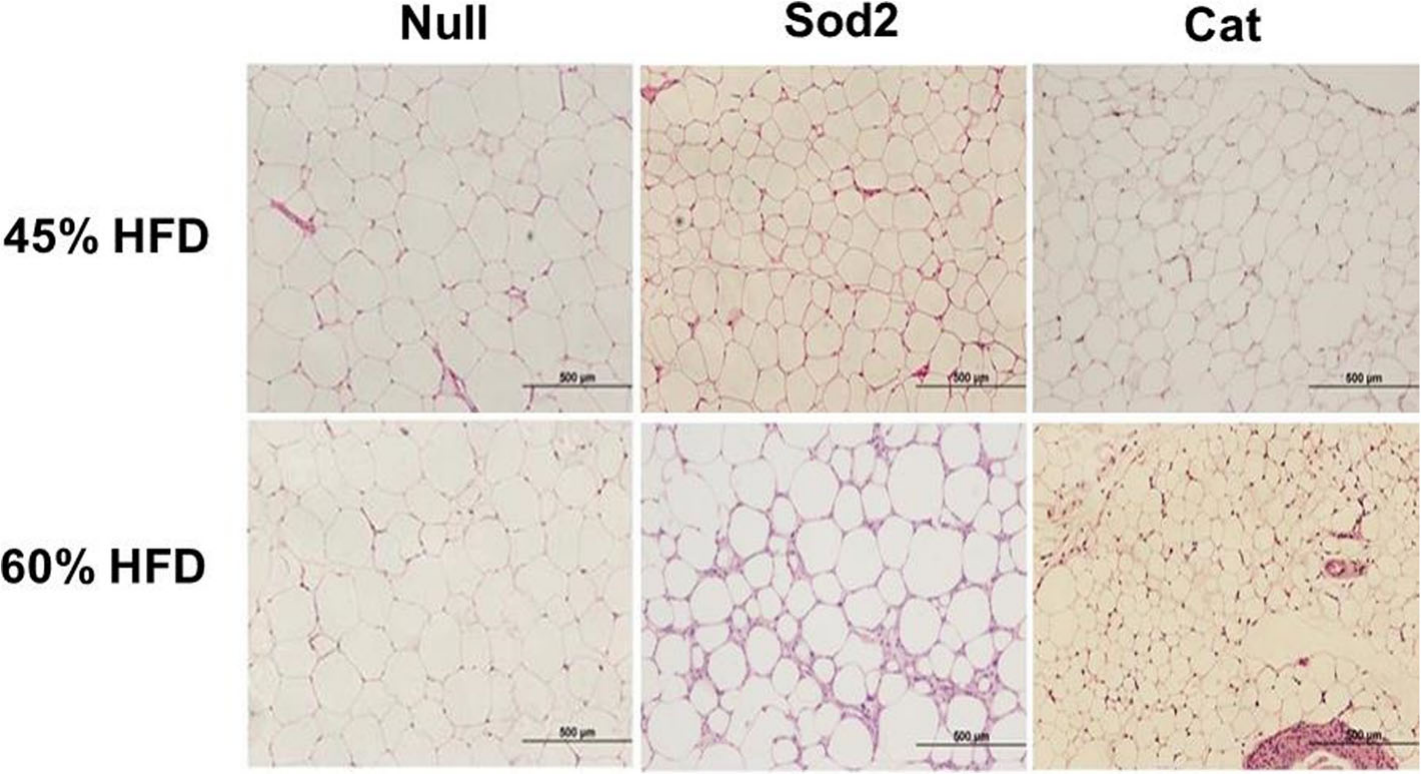
Representative histology images (H&E) of omental fat from DIO mice fed a 45% and 60% HFD. Tissues were harvested at week 4 post-MSC transplantation into DIO mice. Animals that received Sod2- and Cat-MSCs showed less hyperplastic adipocytes as confirmed by cell area measurement (see text)

Interestingly, for omental fat, histological analysis showed less hyperplastic fat cells in the groups that received Sod2- and Cat-MSCs in comparison with Null-MSCs (Fig. 3). Indeed, the cell area of omental fat obtained from animals that were fed a 60% HFD was significantly reduced to approximately half of the size of control samples after MSC therapy as measured by ImageJ program (Null-MSCs = 38,229 μm^2^ ± 2233; Sod2-MSCs = 20,441 μm^2^ ± 2233; Cat-MSCs = 15,319 μm^2^ ± 1641; *p* < 0.001, *n* = 35–50 individual fat cells were measured in each group).

### The effect of modified MSCs on gene expression

Based on the results described above for fluorescence imaging of MSCs, post-transplantation in mice, it indicated “homing in” of the adult adipose-tissue derived adult MSCs into visceral fat depots. We have shown this process to hold true based on our previous study in db/db mice [7]. In addition to less hyperplastic fat cells found in omental fat for mice that received Sod2- and Cat-MSCs, the next step was to investigate the gene of interest expression in the visceral adipose tissue depots. Accordingly, we confirmed that mRNA expression of GOI (Sod2 and Cat) was upregulated in MSCs (in vitro prior to transplantation; Additional file 1: Figure S2), and increase (in vivo) of Sod2 protein expression in omental fat of mice fed with 45% HFD and that received Ad-sod2 transduced MSCs. An estimation of the amount of protein Sod2 detected in omental fat is shown in Additional file 1: Figure S3.

**Fig. 4:**
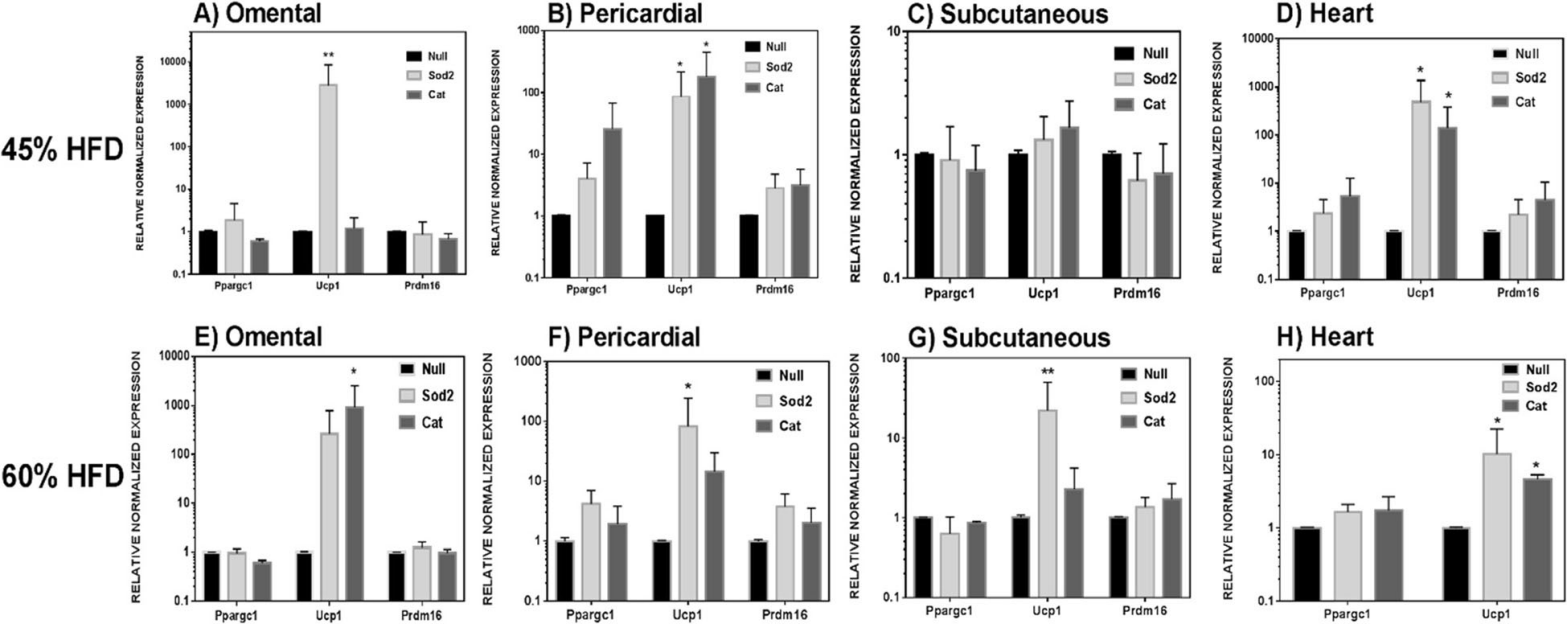
Effect of Sod2- and Cat-MSCs on the gene expression of different tissues from DIO mice fed a 45% HFD and 60% HFD. Fat depots: **a** omental, **b** pericardial, and **c** subcutaneous. **d** Heart. Fat tissues and organs were harvested at week 4 post-MSC transplantation. mRNA expression of UCP1 was predominantly increased in fat depots (**a**–**c**, **e**–**g**) and heart (**d**, **h**). Gene expression was normalized to 18S (*p < 0.05; **p < 0.01), and values are relative to control (Null-MSCs)

**Fig. 5:**
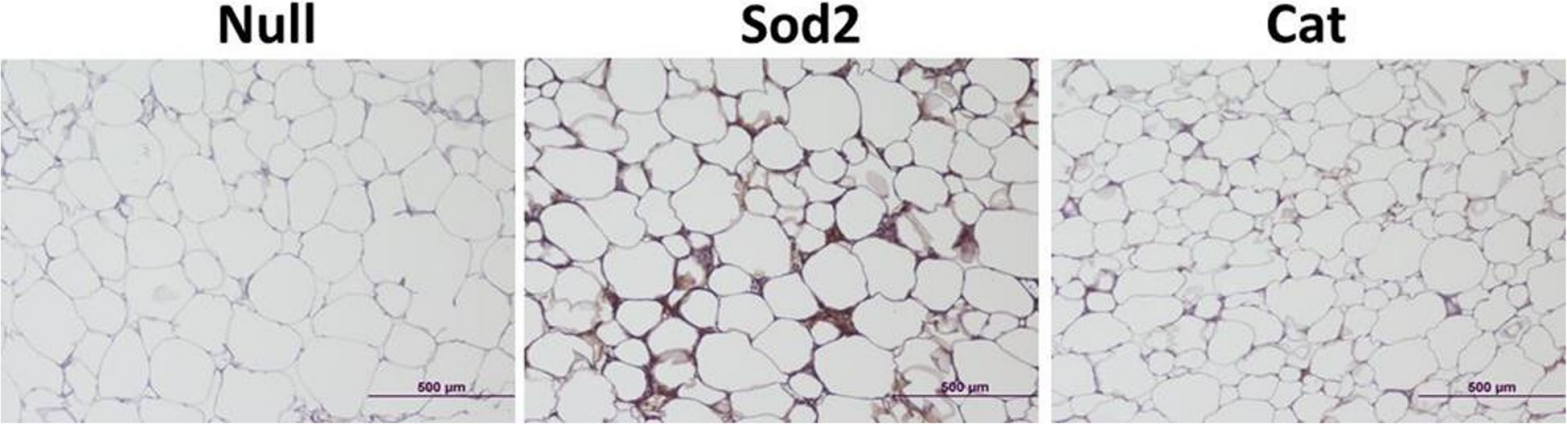
Representative images of Ucp1 immunoreactivity of omental fat from DIO mice fed a 45% HFD. Tissues were harvested at week 4 post-Sod2- and Cat-MSC transplantation. The results indicate a transition from white to brown-like adipose tissue. UCP1-positive staining was nearly twofold by ImageJ analysis for SOD2-receiving cells compared to catalase-receiving cells

Figure 4 shows consistent results for mitochondrial gene array mRNA analysis of fat samples and heart. Ucp1 mRNA upregulation was noted approximately 100–1000-fold for omental mRNA expression and 10–100-fold for pericardial fat, while upregulation for subcutaneous mRNA was 2–20-fold. Upregulation of Ucp1 gene was also noted in the heart of mice that received Sod2- or Cat-MSCs (approximately 10–100-fold) (Fig. 4d, h). The upregulation of UCP1 in omental fat depots was confirmed by immunohistochemistry in mice fed a 45% HFD and that received either Sod2- or Cat-MSCs (Fig. 5). Besides UCP1 mRNA, a trend for PCGC1a and PRDM16 mRNA upregulation was also noted in some of the fat tissues analyzed, particularly in the pericardial fat (Fig. 4b, f) and heart (Fig. 4d). Pericardial fat is known to be metabolically active and may be an important indicator of cardiac health [17–20].

### Modified MSCs promoted a reduction of systemic inflammation

**Fig. 6:**
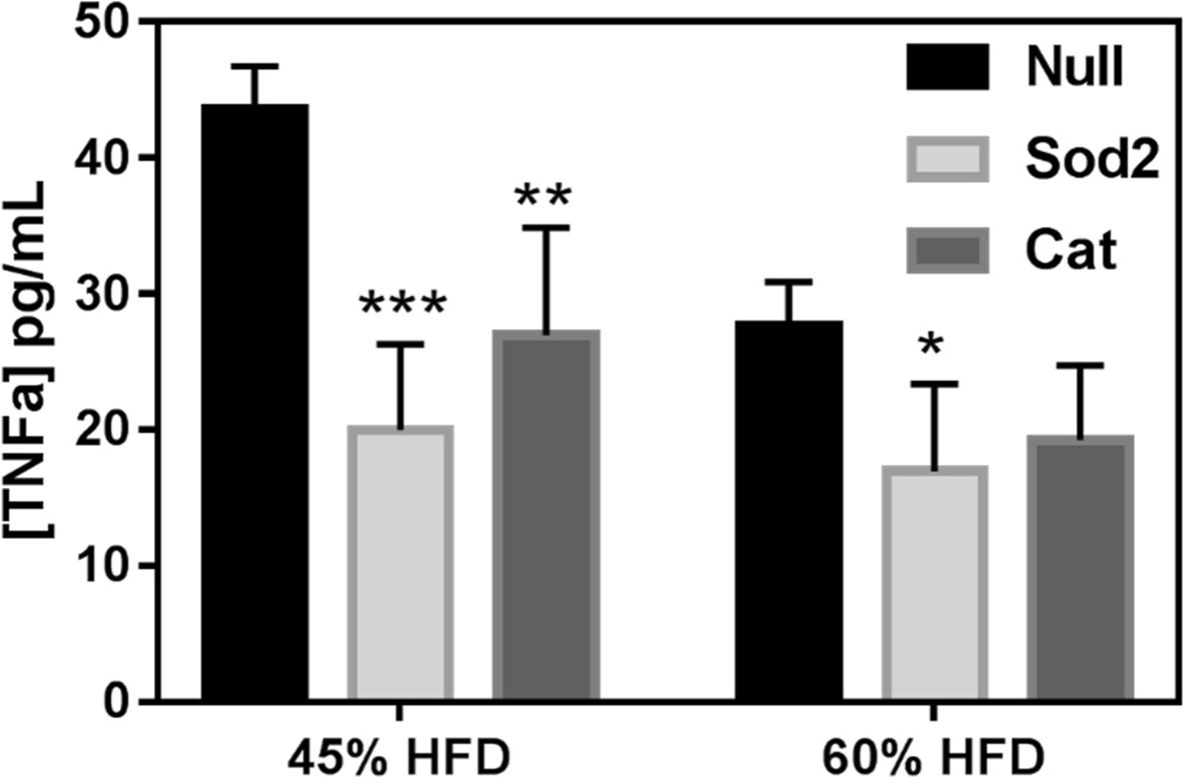
Plasma concentration of inflammatory marker TNFα in mice that were fed either 45% or 60% HFD. Whole blood was collected at week 4 post-MSC transplantation into DIO mice, and plasma was used to perform ELISA assay. Improvement of systemic inflammation was noted in animals that received Sod2- and Cat-MSCs (*p < 0.05, **p < 0.01, ***p < 0.001)

Plasma was obtained at week 4 post-MSC transplantation and used to assess systemic inflammation of the DIO mice. A commercial mouse ELISA kit (Invitrogen, Cat# KMC3011) was used to quantify TNFα. Remarkably, the TNFα values detected in the plasma from mice that received Sod2- and Cat-MSCs were lower than those detected in the control group (Null-MSCs) (Fig. 6). Similar findings were noted for both 45% and 60% HFD.

## Discussion

In our previous publication, we showed that human MSCs exposed to hyperglycemia lead to the accumulation of intracellular reactive oxygen species (ROS). With ROS accumulation, there was an associated mitochondrial dysfunction with complex 1 functional abnormalities. Our previous in vivo experiments using diabetic and obese mouse model (db/db) showed improvement of glucose tolerance on transplanting Sod2-upregulated human MSCs [7].

In our current study, we used two high fat diets, 45% and 60% high-fat diets, for 2–3 months with average body weight of 35–40 g (starting value of approximately 20 g) which is much less than the average weight of 66 g noted for db/db model [7].

As per description of DIO mouse models (The Jackson Laboratory web page), we believe that DIO mice have lesser IR than db/db mice and therefore closer to a prediabetes model rather than mouse model for diabetes. Lesser IR may make the therapy effect of Sod2-MSCs less dramatic in DIO mice compared to db/db mice. In fact, no significant changes between the treated and control groups were noted in the body weight of both DIO mice groups post-antioxidant-upregulated MSC therapy (Additional file 1: Table S1). We chose this model as it is not genetically modified and closer to the human disease of type 2 diabetes which is largely related to poor dietary habits and lifestyle [21].

Similar to our previous study, we have used adenovirus, a DNA virus, as a non-integrating virus (unlike RNA virus that integrates with host genome) to upregulate mouse antioxidants [9, 22]. In this context, the use of AAV, another DNA viral vector [23, 24], to upregulate antioxidants for use in a chronic disease setting of diabetes and obesity may be a good option.

We have upregulated two different antioxidants, one mitochondrial (Sod2) and the other one cytosolic (catalase), individually. Sod2 converts ROS to H_2_O_2_, and catalase helps to convert the latter to water and oxygen [7, 9, 22, 25–27]. The delivery of both antioxidants that were upregulated in MSCs seems to be effective in reducing inflammation and liver fat content. However, mitochondrial Sod2-upregulated MSC cell therapy appears to hold an upper hand in both DIO mouse models (45% and 60% HFD). It is important to note we have upregulated an enzyme rather than a growth factor. Therefore, it is a rate-limiting process that is dependent on the increased substrate presence which in this case is ROS. We believe such a process is safe for possible future human therapeutics. Western blot of Sod2 protein was upregulated in omental fat in animals that received Sod2-upregulated MSCs indicating the local presence of antioxidant.

In our current mouse model of obesity and prediabetes, we noted an improvement of glucose tolerance in the 60% HFD group but to a lesser magnitude than previously described for db/db mice [7]. Interestingly, the differences for area under curve (AUC) between the treatment and control groups for glucose tolerance test (GTT) in mice fed a 45% HFD was less than those observed for the 60% HFD group. This finding is more likely because in these DIO mouse models, mice fed a 45% HFD are less insulin resistant compared to those fed a 60% HFD. Therefore, we believe that the different results found for the DIO mouse models used are related to IR degree. The IR is linked to the total body weight or body fat and indeed visceral fat [21, 25] which in turn is dependent on the diet received; thus, the higher the IR in the model, the better will be the magnitude of the therapeutic response or the delta. As mentioned, IR is also related to fat accumulation in important visceral organs. Indeed, we have noted higher fat accumulation in the liver of mice fed a 60% HFD than mice fed a 45% HFD which incrementally got reduced following modified MSC delivery.

Based on the fluorescence emitted by GFP-expressing MSCs, in both models, we tracked the appropriate homing of MSCs into different fat depots and possibly even the liver up to the time of sacrifice, that is 28 days, shown in Additional file 1. We noted a statistically significant reduction in liver fat accumulation that was confirmed by triglyceride values (see Fig. 2). This is even more clinically important when the weight in our mouse models did not change significantly. Therefore, it appears that Sod2- and Cat-MSC-based cell therapies prevent non-alcoholic fatty infiltration in the liver (NAFLD), independent of weight loss. Treatment of NAFLD is important to prevent progression to NASH and associated permanent damage of the liver. NAFLD is common not only in diabetes but also in prediabetes [21, 26].

Another important biochemical assay other than GTT was the plasma quantification of the pro-inflammatory molecule TNFα which was significantly lower in the treatment groups for both DIO models. Systemic inflammation is a key component of prediabetes and diabetes that is associated with oxidative stress and cardiovascular risk [27]. TNFα plays an important role in mediating inflammatory responses in a state of IR [28]. We believe that the reduction of systemic inflammation by reducing oxidative stress played a key role in improving the fatty infiltration of the liver, independent of weight reduction [29].

Along with inflammation reduction, another factor that most likely played a role in improving NAFLD is browning/beiging of visceral fat particularly in omental and pericardial fat depots. Functional improvement of pericardial fat may help reduce CVD risk in metabolically diseased models of prediabetes and diabetes. Ucp1, Pgc1a, and Prdm16 upregulation was tested by RT-PCR in the omental, pericardial, and subcutaneous fat and even the heart. All of these tested tissues showed Ucp1 upregulation, consistently more so in the 60% DIO model compared to the 45% DIO model. Interestingly, energy-efficient tissues such as the pericardial fat and heart consistently showed concomitant Pgc1a upregulation also. We believe that improvement in mitochondrial function and browning (evidenced by UCP1 and PGC1A upregulation) of white fat may have helped to reduce the systemic inflammation (as seen with TNFα plasma values reduction; Fig. 6) with subsequent ameliorating of fatty liver disease [30]. It appears the Ucp1 upregulation is more prominent in fat depots and heart in the animals that received -Sod2 upregulated MSC- compared to catalase-upregulated MSC-receiving mice. Our Ucp1 staining figure also indicates a similar outcome.

At this point, our conclusion is that the improvements in liver fat accumulation and glucose tolerance in DIO mouse models are secondary to a combination effect of reduction in systemic inflammation and an increase in energy efficiency by upregulating Ucp1 in white fat depots.

Whether systemic inflammation drives beiging/browning or browning drives reduction in systemic inflammation which subsequently improves NAFLD is an important and clinically relevant question that needs to be addressed.

In our DIO mouse models, we have shown that the use of antioxidant-upregulated MSCs (used as a cell delivery vehicle for GOI) delivered intraperitoneally increases antioxidant presence in the intra-abdominal areas such as omental fat and ameliorates a prevalent metabolic syndrome complication such as fatty liver disease by promoting browning of white fat and more importantly reducing systemic inflammation.

In conclusion, we have demonstrated that antioxidant upregulated adipose tissue derived MSC delivery can be a safe yet effective therapy for NAFLD in the mouse model of diet-induced obesity and prediabetes.

## Additional file

Additional file 1: **Figure S1.** Changes in body weight of DIO mice fed high-fat diets (45% and 60% HFD) prior to MSCs transplantation (*n*= 10 each group). Arrows indicate time point that animals received MSCs. **Table S1.** Body weight of mice prior and post MSCs injection: Null-MSCs, Sod2-MSCs and Cat-MSCs. **Figure S2.** Upregulation of Sod2 and Cat in fat-derived MSCs transduced with adenovirus. After cells were transduced with Ad-Sod2-GFP, Ad-Cat-GFP, and Ad-Null-GFP, MSCs were then cultured in adipogenic media (Lonza) in alternated cycles of 3 days of induction and 1 day of maintenance (3-cycles total). The results correspond to two independent experiments, *p<0.05 (multiple t-tests corrected for multiple comparisons using the Holm-Sidak method). Gene expression was normalized to 18S and values are relative to control (Null-MSCs). **Figure S3.** A) Representative western blot image showing the presence of Sod2 and beta-actin (control) in omental fat from 45% HFD mice which received Null-MSCs (lane 1) and Sod2-MSCs (lane 2). B) Relative quantification of the Sod2 bands showed in A) was performed with ImageJ using beta-actin as a loading control. The results are shown as Sod2/beta-actin ratio and indicated higher amounts of Sod2 in the fat depots for mouse that received Sod2-MSCs (*n*=2). **Figure S4.** Representative image of DIO mice that received 1.5 x 10^6^ MSCs previously transduced with AdNull (control), AdSod2, and AdCat, Cells were intraperitoneally transplanted into DIO mice and monitored for 28 days. Longitudinal whole-body fluorescent imaging of mice fed a 60% HFD demonstrates the feasibility of tracking MSCs over time. The imaging system indicates possible MSCs homing to different adipose tissue regions and prominent florescence at day 7 with persistence of signal at day 28 (at the time of sacrifice), post MSC delivery. (DOCX 1488 kb)

## Data Availability

The datasets used and/or analyzed during the current study are available from the corresponding author on reasonable request.

## Abbreviations

Ad: Adenovirus
Cat: Catalase
CVD: Cardiovascular diseases
DIO: Diet-induced obese
DMEM: Dulbecco’s modified Eagle’s medium
ELISA: Enzyme-linked immunosorbent assay
FBS: Fetal bovine serum
GFP: Green fluorescent protein
GOI: Gene of interest
GTT: Glucose tolerance test
H&E: Hematoxylin and eosin
HFD: High-fat diet
IP: Intraperitoneally
IR: Insulin resistance
MOI: Multiplicity of infection
MSCs: Mesenchymal stem cell
NAFLD: Non-alcoholic fatty liver disease
NASH: Non-alcoholic steatohepatitis
Ppargc1a: Peroxisome proliferative activated receptor, gamma, coactivator 1 alpha
Prdm16: PR domain containing 16
ROS: Reactive oxygen species
Sod2: Superoxide dismutase 2
T2D: Type 2 diabetes
TNFα: Tumor necrosis factor alpha
UCP1: Uncoupling protein 1
