# Supplementary material for: Antioxidant-upregulated mesenchymal stem cells reduce inflammation and improve fatty liver disease in diet-induced obesity
Source: Stem Cell Res Ther. 2019 Sep 2;10:280. doi: 10.1186/s13287-019-1393-8 (PMC6720095; doi:10.1186/s13287-019-1393-8)
Supplement: Supplementary file 1 — Figure S1. Changes in body weight of DIO mice fed high-fat diets (45% and 60% HFD) prior to MSCs transplantation (n= 10 each group). Arrows indicate time point that animals received MSCs. Table S1. Body weight of mice prior and post MSCs injection: Null-MSCs, Sod2-MSCs and Cat-MSCs. Figure S2. Upregulation of Sod2 and Cat in fat-derived MSCs transduced with adenovirus. After cells were transduced with Ad-Sod2-GFP, Ad-Cat-GFP, and Ad-Null-GFP, MSCs were then cultured in adipogenic media (Lonza) in alternated cycles of 3 days of induction and 1 day of maintenance (3-cycles total). The results correspond to two independent experiments, *p<0.05 (multiple t-tests corrected for multiple comparisons using the Holm-Sidak method). Gene expression was normalized to 18S and values are relative to control (Null-MSCs). Figure S3. A) Representative western blot image showing the presence of Sod2 and beta-actin (control) in omental fat from 45% HFD mice which received Null-MSCs (lane 1) and Sod2-MSCs (lane 2). B) Relative quantification of the Sod2 bands showed in A) was performed with ImageJ using beta-actin as a loading control. The results are shown as Sod2/beta-actin ratio and indicated higher amounts of Sod2 in the fat depots for mouse that received Sod2-MSCs (n=2). Figure S4. Representative image of DIO mice that received 1.5 x 10^6^ MSCs previously transduced with AdNull (control), AdSod2, and AdCat, Cells were intraperitoneally transplanted into DIO mice and monitored for 28 days. Longitudinal whole-body fluorescent imaging of mice fed a 60% HFD demonstrates the feasibility of tracking MSCs over time. The imaging system indicates possible MSCs homing to different adipose tissue regions and prominent florescence at day 7 with persistence of signal at day 28 (at the time of sacrifice), post MSC delivery. (DOCX 1488 kb) [file 13287_2019_1393_MOESM1_ESM.docx]

**
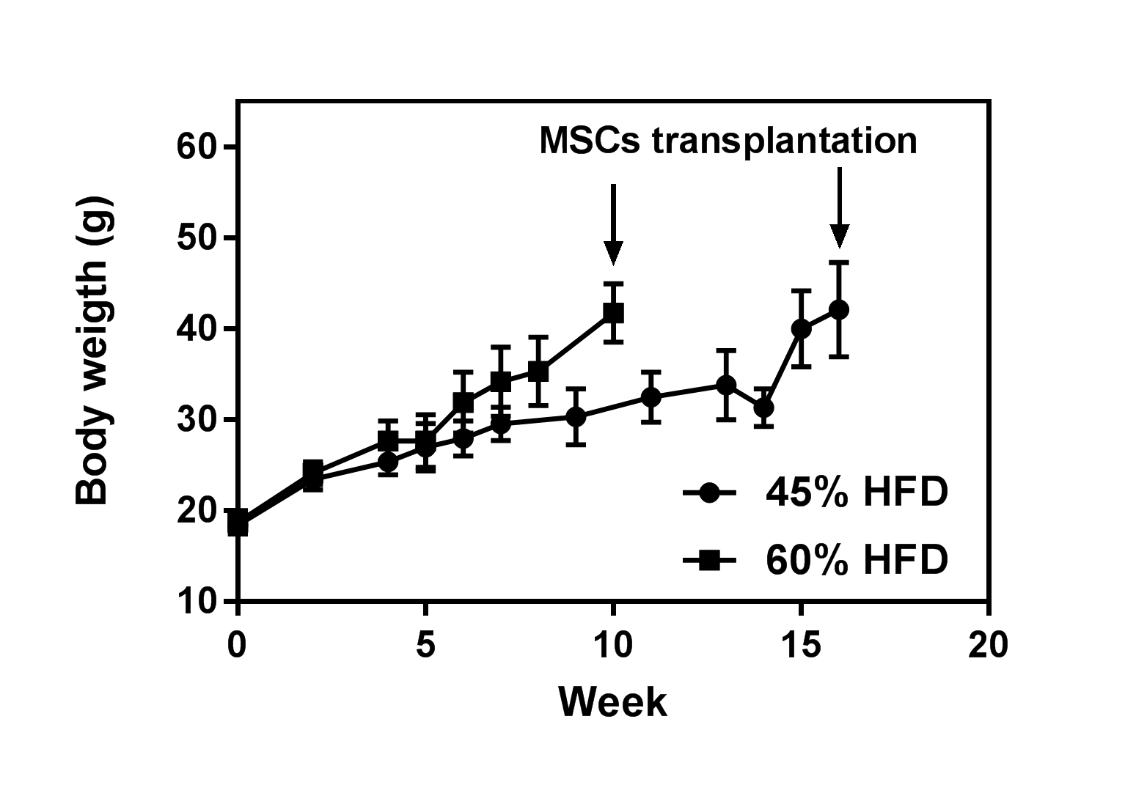
**

**Figure S1** – Changes in body weight of DIO mice fed high-fat diets (45% and 60% HFD) prior to MSCs transplantation (n= 10 each group). Arrows indicate time point that animals received MSCs.

**Table S1** – Body weight of mice prior and post MSCs injection: Null-MSCs, Sod2-MSCs and Cat-MSCs.

| **Time** | **45% HFD** | | | **60% HFD** | | |
| --- | --- | --- | --- | --- | --- | --- |
|  | **Null** | **Sod2** | **Cat** | **Null** | **Sod2** | **Cat** |
| **Week 0** | 35.6 ± 7 | 36.7 ± 6.3 | 39 ± 7.2 | 41.5 ± 9 | 41.3 ± 3.7 | 36.6 ± 5.9 |
| **Week 2** | 36 ± 8.1 | 37 ± 7 | 40 ± 7.5 | 40.3 ± 6.6 | 40.5 ± 7.8 | 38 ± 7 |
| **Week 4** | 36 ± 8.1 | 37.2 ± 8.5 | 40.6 ± 7 | 41.6 ± 7.5 | 43.2 ± 5.7 | 39.6 ± 7.5 |

**
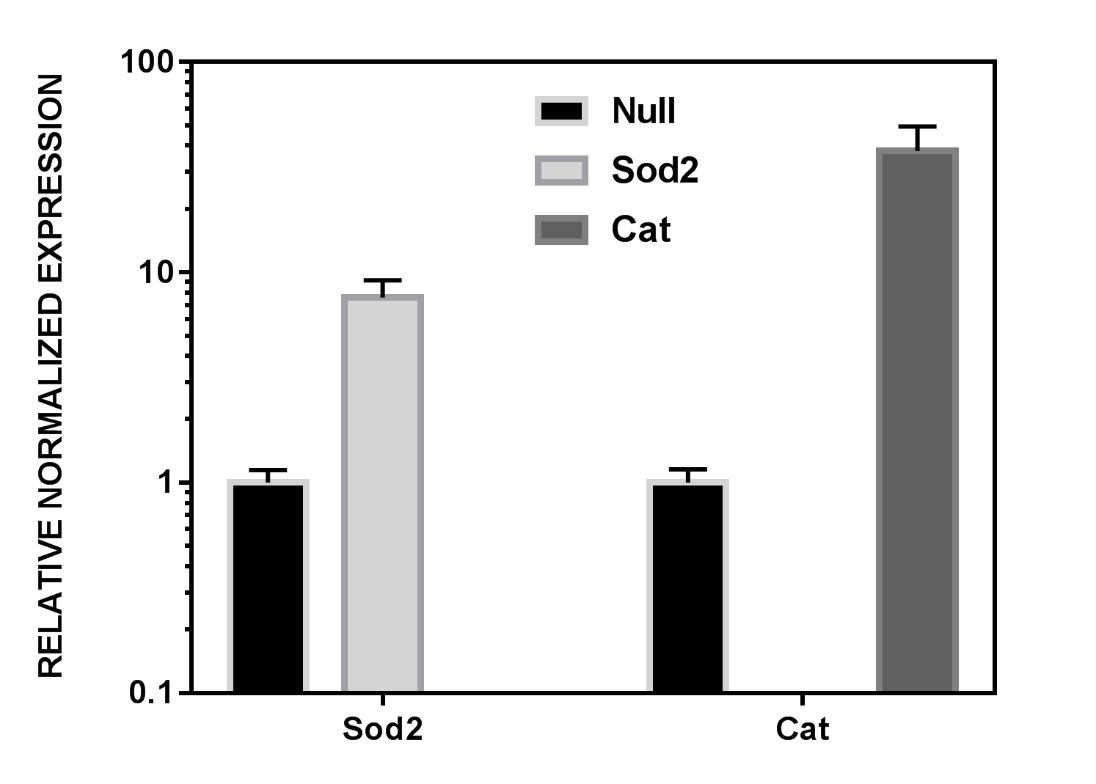
**

**Figure S2 -** Upregulation of Sod2 and Cat in fat-derived MSCs transduced with adenovirus. After cells were transduced with Ad-Sod2-GFP, Ad-Cat-GFP, and Ad-Null-GFP, MSCs were then cultured in adipogenic media (Lonza) in alternated cycles of 3 days of induction and 1 day of maintenance (3-cycles total). The results correspond to two independent experiments, *p<0.05 (multiple t-tests corrected for multiple comparisons using the Holm-Sidak method). Gene expression was normalized to 18S and values are relative to control (Null-MSCs).

**
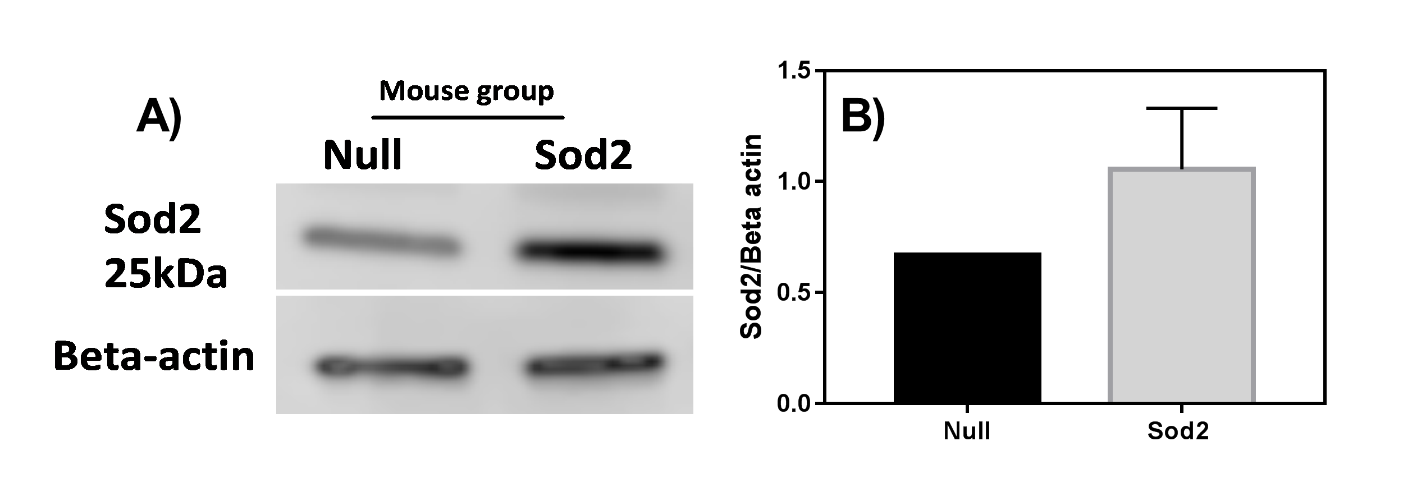
**

**Figure S3** – A) Representative western blot image showing the presence of Sod2 and beta-actin (control) in **omental fat from 45% HFD mice** which received Null-MSCs (lane 1) and Sod2-MSCs (lane 2). B) Relative quantification of the Sod2 bands showed in A) was performed with ImageJ using beta-actin as a loading control. The results are shown as Sod2/beta-actin ratio and indicated higher amounts of Sod2 in the fat depots for mouse that received Sod2-MSCs (n=2).


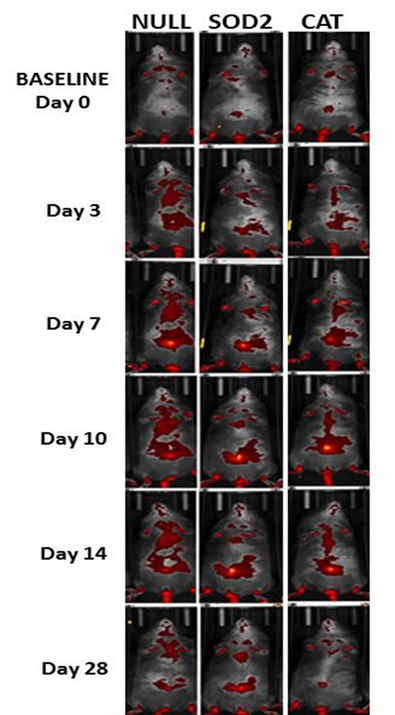

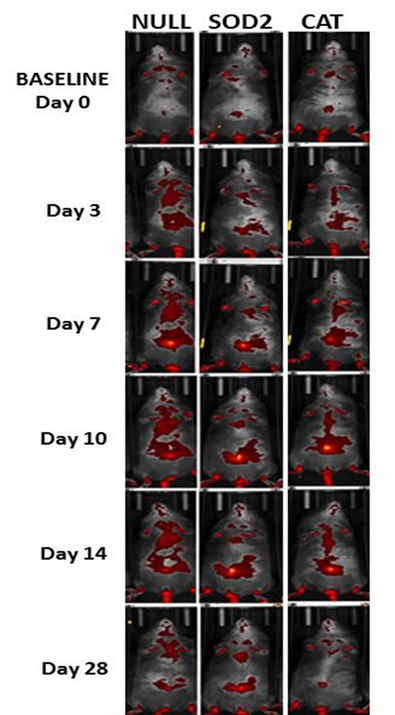

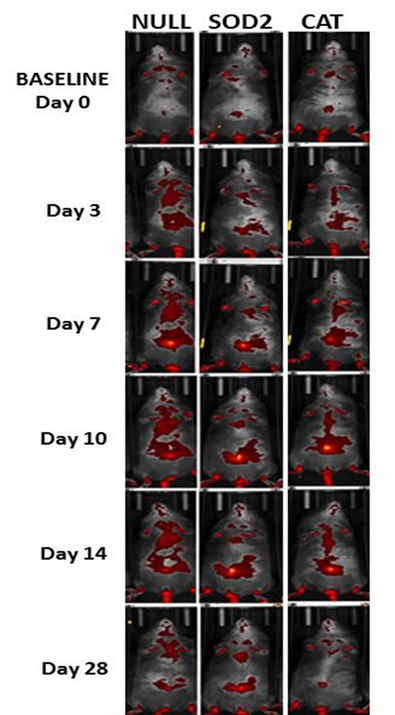


**Figure S4:** Representative image of DIO mice that received 1.5 x 10^6^ MSCs previously transduced with AdNull (control), AdSod2, and AdCat, Cells were intraperitoneally transplanted into DIO mice and monitored for 28 days. Longitudinal whole-body fluorescent imaging of mice fed a 60% HFD demonstrates the feasibility of tracking MSCs over time. The imaging system indicates possible MSCs homing to different adipose tissue regions and prominent florescence at day 7 with persistence of signal at day 28 (at the time of sacrifice), post MSC delivery
